# Myeloid-specific expression of Stat3C results in conversion of bone marrow mesenchymal stem cells into alveolar type II epithelial cells in the lung

**DOI:** 10.1007/s11427-012-4339-2

**Published:** 2012-08-04

**Authors:** YAN Cong, QU Peng, DU Hong

**Affiliations:** 1 Department of Pathology and Laboratory Medicine, Indiana University School of Medicine, Indianapolis, IN 46202-5188, USA; 2 The Center for Immunobiology, Indiana University School of Medicine, Indianapolis, IN 46202-5188, USA; 3 IU Simon Cancer Center, Indiana University School of Medicine, Indianapolis, IN 46202-5188, USA

**Keywords:** Stat3C, mesenchymal stem cells, lung epithelial cells, transgenic mice, tissue remodeling, myeloid-derived suppressive cells (MDSCs)

## Abstract

Bone marrow mesenchymal stem cells (BMSCs) and myeloid lineage cells originate from the bone marrow, and influence each other *in vivo*. To elucidate the mechanism that controls the interrelationship between these two cell types, the signaling pathway of signal transducer and activator of transcription 3 (Stat3) was activated by overexpressing Stat3C in a newly established c-fms-rtTA/(TetO)_7_-CMV-Stat3C bitransgenic mouse model. In this system, Stat3C-Flag fusion protein was overexpressed in myeloid lineage cells after doxycycline treatment. Stat3C overexpression induced systematic elevation of macrophages and neutrophils in multiple organs. In the lung, tissue neoplastic pneumocyte proliferation was observed. After *in vitro* cultured hSP-B 1.5-kb lacZ BMSCs were injected into the bitransgenic mice, BMSCs were able to repopulate in multiple organs, self-renew in the bone marrow and spleen, and convert into alveolar type II epithelial cells. The bone marrow transplantation study indicated that increases of myeloid lineage cells and BMSC-AT II cell conversion were due to malfunction of myeloid progenitor cells as a result of Stat3C overexpression. The study supports the concept that activation of the Stat3 pathway in myeloid cells plays an important role in BMSC function, including homing, repopulating and converting into residential AT II epithelial cells in the lung.

The lung has the largest epithelial surface area of the body in order to facilitate air exchange. Alveolar type II (AT II) epithelial cells synthesize and secrete pulmonary surfactant that is critical to prevent alveoli from collapse during respiratory cycles [1,2]. AT II epithelial cells also participate in pulmonary inflammation and injury repair. In the lung, AT II epithelial cells are the local progenitor cells for the alveoli and undergo significant phenotypic changes to terminally differentiate into AT I cells [3,4]. In response to injury, AT II epithelial cells proliferate to create more AT II epithelial cells and differentiate into AT I epithelial cells [5]. Since the lung is constantly exposed to a range of insults from the surrounding environment, maintenance of lung function requires both host defense and epithelial cell damage repair. Given the importance of AT II epithelial cells in the lung, it is necessary to understand homeostasis of AT II epithelial cells under disease conditions.

Bone marrow stem cells (BMSCs) have multiple potentials to convert into residential cells in multiple organs. They can be used for regenerative medicine, tissue repair and gene therapy. Extensive studies demonstrated that BMSCs contribute to lung tissue repairing during damage in various conditions [6]. However, the molecular pathway and the genetic network that control these processes are poorly understood. The lung depends on pulmonary surfactant for the normal respiratory and host defense functions. We demonstrated that blockage of neutral lipid metabolism induced BMSC conversion into AT II epithelial cells as a result of pulmonary remodeling (emphysema and hypercellularity) in lysosomal acid lipase (LAL) knock-out mice (*lal*^−/−^) [7]. In *lal*^−/−^ mice, BMSC-AT II cell conversion is highly associated with systemic inflammation, especially with infiltration of myeloid cells (e.g., myeloid-derived suppressive cells) into the lung. These studies suggest a tight connection between functions of myeloid cells and BMSCs that are controlled by neutral lipid derivative hormones and downstream mediators. Importantly, correction of LAL expression in myeloid cells alone significantly improved systemic inflammation and tissue remodeling [8,9]. Overexpression of LAL downstream genes in myeloid cells induced systemic inflammation and tumorigenesis in the lung [10–12]. Because both myeloid cells and BMSCs are originated from the bone marrow, we hypothesize that malfunction of myeloid cells may affect the functional role of BMSCs *in vivo*. We aim at identifying pro-inflammatory intracellular signaling molecules in myeloid cells that control migration, repopulation and conversion of BMSCs to AT II epithelial cells *in vivo*.

One such molecule is signal transducer and activator of transcription 3 (Stat3). Stat3 was originally identified as the acute phase response factor [13–15]. It is mainly activated by pro-inflammatory interleukin 6 (IL-6) family cytokines that share the common gp130 receptor subunit [16,17]. We demonstrated that persistent activation of the Stat3 pathway in lung epithelial cells directly caused pulmonary inflammation and bronchioalveolar adenocarcinoma [18,19], supporting the concept that Stat3 is a pro-inflammatory molecule and oncogene [20]. It has been reported that Stat3 was involved in myeloid differentiation [21]. Activation of Stat3 by phosphorylation at Y705 was observed in myeloid cells of *lal*^−/−^ mice (Figure 1). To elucidate how activation of the Stat3 pathway in myeloid cells influences repopulation and AT II epithelial cell conversion of BMSCs, a previously established myeloid-specific doxycycline-inducible bitransgenic mouse model system [8] was used to specifically induce Stat3C (a constitutively active form of Stat3) overexpression. In this system, the “activator” transgenic mouse line bears the reverse tetracycline-responsive transactivator (rtTA) fusion protein under the control of the 7.2 kb 5′-flanking regulatory sequence and the downstream intron 2 of the c-fms gene [22] (designated as c-fms-rtTA mice). The rtTA expression is restricted to myeloid lineage cells in transgenic mice. In the second transgenic mouse line, the Stat3C cDNA is under the control of the tet operator DNA binding sequence that is linked to a minimal promoter (designated as (TetO)_7_-CMV-Stat3C mice) [18,23]. After cross-breeding, expression of Stat3C is induced by addition of doxycycline in bitransgenic mice (designated as c-fms-rtTA/(TetO)_7_-CMV-Stat3C bitransgenic mice). We discovered that activation of the Stat3 pathway in myeloid cells of c-fms-rtTA/(TetO)_7_-CMV-Stat3C bitransgenic mice caused systemic increase of myeloid-derived suppressive cells (MDSCs) from the hematopoietic system (e.g., bone marrow and blood) to the immune system (e.g., spleen) to the tissue organs (e.g., lung). By using hSP-B 1.5-kb lacZ adherent BMSCs, we demonstrated that Stat3C overexpression in myeloid cells facilitates migration and repopulation of BMSCs in the bone marrow and spleen of c-fms-rtTA/(TetO)_7_-CMV-Stat3C recipient bitransgenic mice. It also facilitates BMSC-AT II cell conversion at a 10% rate in doxycycline-treated recipient c-fms-rtTA/(TetO)_7_-CMV-Stat3C bitransgenic mice. The study supports the concept that activation of the Stat3 pathway in myeloid cells can facilitate BMSC residing in the recipient mouse bone marrow, migrating into the lung and converting into residential AT II epithelial cells.

## 1 Materials and methods

### 1.1 Animal care

All scientific protocols involving the use of animals in this study have been approved by the Institution Animal Care and Usage Committee (IACUC) and follow guidelines established by the Panel on Euthanasia of the American Veterinary Medical Association. Protocols involving the use of recombinant DNA or biohazardous materials have been reviewed by the Biosafety Committee, and followed the guidelines established by the NIH. Animals were housed under IACUC-approved conditions in a secured animal facility. Animals were regularly screened for common pathogens (specific pathogen free). Experiments involving animal sacrifice utilize CO_2_ narcosis to minimize animal discomfort.

### 1.2 Generation of doxycycline-controlled Stat3 transgenic mice

The (TetO)_7_-CMV-Stat3C transgenic mouse line and the c-fms-rtTA transgenic mice were established as previously described [8,23]. C-fms-rtTA/(TetO)_7_-CMV-Stat3C bitransgenic mice were obtained by crossbreeding the c-fms-rtTA transgenic mice and (TetO)_7_-CMV- Stat3C transgenic mice. In general, animals were treated with doxycycline at 1 month old age after genotyping.

### 1.3 AT II cell and macrophage purification

Alveolar type II epithelial cells and macrophages were purified as described previously [7].

### 1.4 BMSCs isolation, culturing and marker defining

The procedures for BMSC isolation and *in vitro* culturing were the same as previously described [7].

### 1.5 FACS analyses

FACS analysis of CD11b, Gr-1, CD3 and B220 cells was the same as previously described [24,25]. FACS analysis of Stat3C-Flag fusion protein was analyzed by monoclonal ANTI-FLAG^®^ M2-FITC antibody (F4049; Sigma, Saint Louis, MI). For intracellular phosphor-Stat3 analysis in *lal*^−/−^ mice, the assay was performed according to the protocols from Cell Signaling Technology (Danvers, MA). Briefly, after cell surface marker staining, cell suspensions from bone marrow, blood, spleen and lung of *lal*^−/−^ mice were fixed with 2% formaldehyde. Fixation samples were resuspended in methanol at a final 90% concentration. Finally, samples were washed and resuspended in 1× phosphate-buffered saline (PBS) containing 4% fetal bovine serum (FBS) at 10^6^ cells in 100 μL. Cell suspensions were labeled with the primary anti-phospho-Stat3 antibody (Cell Signaling Technology) for 30 min at room temperature, washed in PBS containing 4% FBS, and labeled using the secondary antibody. After washing, samples were analyzed by FACS.

For BMSC marker analysis, *in vitro* cultured BMSCs (1×10^6^–2×10^6^ cells) at passages 0, 5, 7, 9, 11, 13, 15, 17, 19 were harvested and blocked with FcR followed by incubation with primary antibodies in FACS buffer. Anti-SSEA-1 (Abcam, MA), anti-CD106 (VCAM-1; eBioscience), anti-CD44 (IM7; eBiosciences), anti-Api6 (provided by Dr. Miyazaki, The University of Tokyo, Japan), anti-OCT-3/4 (Octamer-3/4, N19; Santa Cruz Biotechnology, Santa Cruz, CA), anti-Nanog (Millipore, Temecula, CA), anti-BMP4 (Bone morphogenic protein-4; Santa Cruz Biotechnology, CA), anti-SOX2 (SRY, or sex determining region Y-box 2; R&D Systems, Inc., Minneapolis, MN), anti-C-kit (CD117; eBiosciences), anti-Sca-1 (Ly6A/E, Stem Cell Antigen 1; eBiosciences), a biotin-Conjugated Mouse Lineage Panel (including biotin anti-mouse CD3ε, CD11b (Mac-1), CD45R/B220, TER-119, Ly-6G and Ly-6C; BD Biosciences, San Jose, CA) and AT II epithelial marker SP-C (FL-197; Santa Cruz Biotechnology, Santa Cruz, CA) were purchased for FACS analyses on a LSRII machine (BD Biosciences, San Jose, CA). Data was analyzed using the BD FACStation™ Software (BD Biosciences).

For the BMSC-AT II cell conversion study, AT II epithelial cells were labeled with PE anti-SP-C and FITC anti-β-galactosidase antibodies and analyzed as previously described [7]. Isotype controls IgG1, IgG2a and IgG2b were included in all experiments. Quadrants were assigned using isotype control.

### 1.6 BMSC *in vivo* tracing and proliferation study

The procedure was performed essentially the same as we described previously [7], except that c-fms-rtTA/(TetO)_7_-CMV-Stat3C bitransgenic mice were used.

### 1.7 Real-time PCR

For Real-time PCR analysis of the lacZ gene, total RNAs were purified from AT II epithelial cells using Qiagen RNA purification kit. A pair of sequence-specific oligonucleotide primers for the lacZ gene was used. Glyceraldehyde 3-phosphate (GAPDH) primers were used as an endogenous control and normalizer for all samples.

### 1.8 Double immunofluorescence staining

The procedure for isolation and immunofluorescence staining of AT II epithelial cells was the same as previously described [7]. For lung tissue section staining, tissue sections were rinsed in PBS-Tween 20 for 5 min, and incubated with normal mouse serum for 30 min to block non-specific binding of immunoglobulin. The sections were double stained with SP-C rabbit antibody (Santa Cruz Biotechnology, CA) and β-galactosidase mouse antibody (1:100 Sigma, St Louis, MO, USA). A Cy2-conjugated donkey anti-rabbit IgG and a Cy3-conjugated donkey anti-mouse IgG (Jackson ImmunoResearch, West Grove, PA, USA) were used as the secondary antibodies.

### 1.9 Immunoelectron microscopy

For post-embedding electron microscopy, lung tissues were fixed with 4% paraformaldehyde in 0.1 mol L^−1^ phosphate buffer, dehydrated through a graded series of ethyl alcohols and embedded in Unicryl (Electron Microscopy Sciences, Hatfield, PA). Thin sections (70–90 nm) were mounted on Formvar/carbon coated nickel grids. The grids were placed into a blocking buffer for 60 min and then into the primary antibody overnight at 4°C. The grids were then rinsed with phosphate buffered saline (PBS) and floated on drops of the appropriate secondary antibody attached with 10 nm gold particles (AURION, Hatfield, PA) for 2 h at room temperature. After rinsing with PBS the grids were placed in 2.5% glutaraldehyde in 0.1 mol L^−1^ phosphate buffer for 15 min. After rinses with PBS and distilled water, the grids were allowed to dry and then stained for contrast with uranyl acetate. The samples were viewed with a Tecnai Bio Twin transmission electron microscope (FEI, Hillsboro, OR) and images taken with a CCD camera (Advanced Microscopy Techniques, Danvers, MA). The experiment was performed by Electron Microscopy Core Facility at Indiana University School of Medicine.

### 1.10 Bone marrow transplantation

The procedure for bone marrow transplantation experiment was similar to that previously described [26]. Briefly, recipient wild type mice (WT, CD45.2^+^) at 3-week old were lethally irradiated with 1000 rad of γ-irradiation and rested 1 d prior to receiving cells. Bone marrow cells were prepared from femurs and tibia of 8−10-week old donor wild type or c-fms-rtTA/(TetO)_7_-CMV-Stat3C bitransgenic mice (CD45.1^+^). Reciprocal recipient mice (CD45.2^+^) were injected with 2.5×10^6^–5×10^6^ donor bone marrow cells in 500 μL 1× PBS via tail vein. The chimeric mice either were treated or untreated with doxycycline for three months. A group of these chimeric mice were used for inflammatory cell analyses. Another group of these chimeric mice were used for the BMSC injection study. Cultured adherent hSP-B 1.5-kb lacZ BMSCs after 9 passages were harvested and resuspended in 1× PBS. Each recipient chimeric mouse was infused with adherent hSP-B 1.5-kb lacZ BMSCs through tail vein as described above. After 6 weeks of BMSCs injection, the bone marrow, blood, spleen and lungs from various groups were harvested for BMSC-AT II cell conversion analysis.

### 1.11 Statistical analysis

The data shown were mean values of at least three independent experiments and expressed as mean±SD. A paired Student’s *t* test or ANOVA was used to evaluate the significance of the differences. Statistical significance was set at a level of *P*<0.05.

## 2 Results

### 2.1 Activation of Stat3 in myeloid cells during LAL deficiency

**Figure 1:**
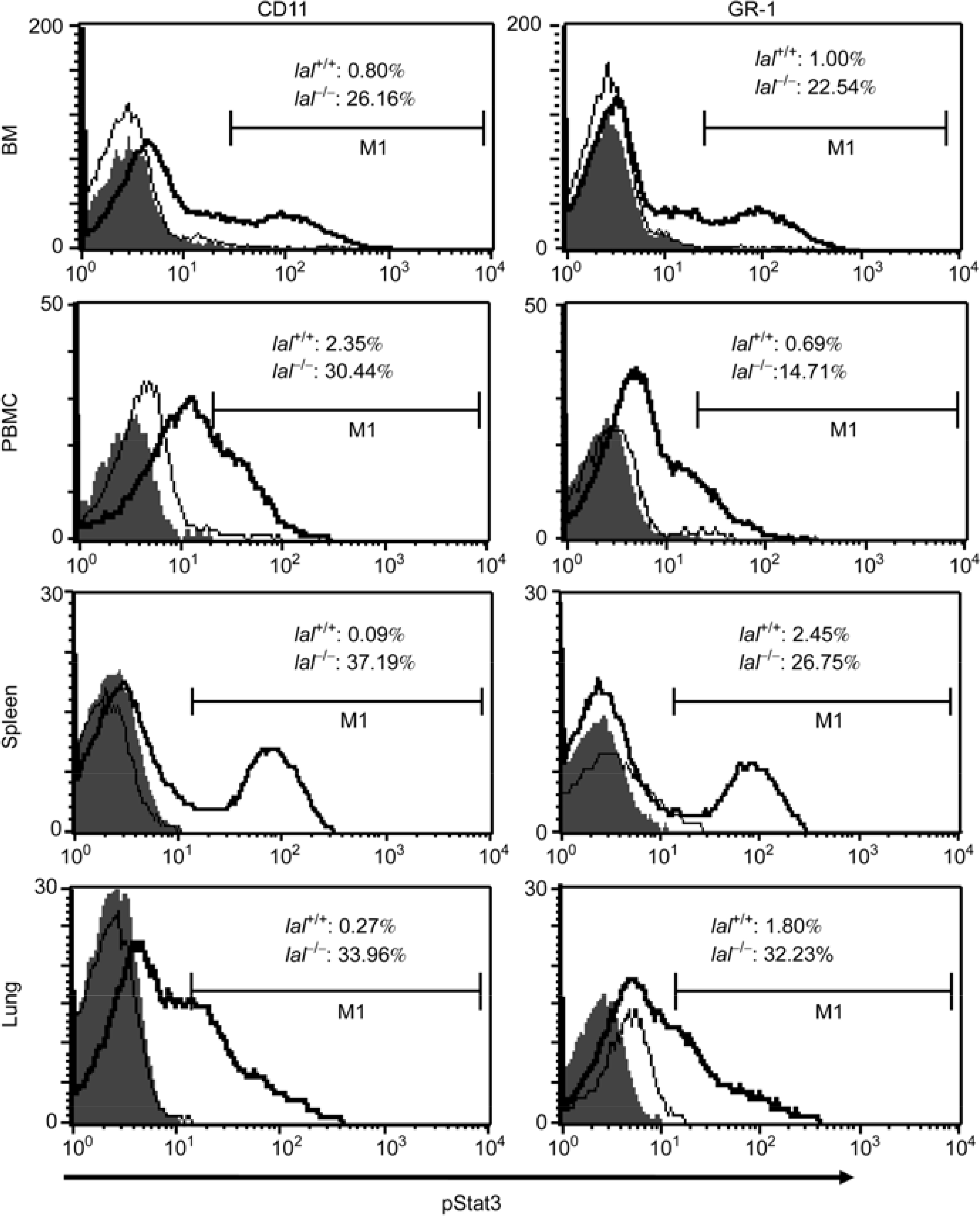
Activation of Stat3 in myeloid lineage cells in *lal*^−/−^ mice. Single cell suspensions were prepared from the bone marrow, blood (PBMC), spleen and lung of 3-month *lal*^+/+^ and *lal*^−/−^ mice for FACS analyses of relative phosphor-Stat3 cell populations (pStat3) in myeloid cells. Each experiment represents 3 independent (*n*=3) studies. *lal*^+/+^, wild-type mice (gray line); *lal*^−/−^, LAL knockout mice (black line); Shaded areas were isotype controls. M1 is a histogram marker excluding isotypic control (negative control).

During LAL deficiency, systemic infiltration of myeloid-lineage macrophages and neutrophils in multiple organs is a major manifestation of pathogenesis [8,27–29]. Inflammation triggered BMSC conversion into AT II epithelial cells in *lal*^−/−^ mice [7]. However, the cellular and molecular mechanism for this event is not clear. Because both myeloid lineage cells and BMSCs are originated from the bone marrow, it is possible that activation of myeloid lineage cells affects the BMSC behavior. To identify which inflammatory signaling molecules are activated in myeloid-lineage cells during LAL deficiency, important intracellular signaling molecules were tested by FACS. Interestingly, activation of Stat3 phosphorylation at Y705 was observed at various developmental stages of myeloid-lineage cells in multiple organs, including the bone marrow, blood, spleen and lung (Figure 1). Our results demonstrated that the percentage numbers of phosphor-Stat3 positive CD11b^+^ macrophages and GR-1^+^ neutrophils in *lal*^−/−^ mice were highly increased compared with those of *lal*^*+/+*^ mice. Therefore, it is possible that Stat3 activation in myeloid lineage cells facilitate BMSCs migration, homing and converting into AT II epithelial cells.

### 2.2 Generation of c-fms-rtTA/(TetO)_7_-CMV-Stat3 bitransgenic mice

**Figure 2:**
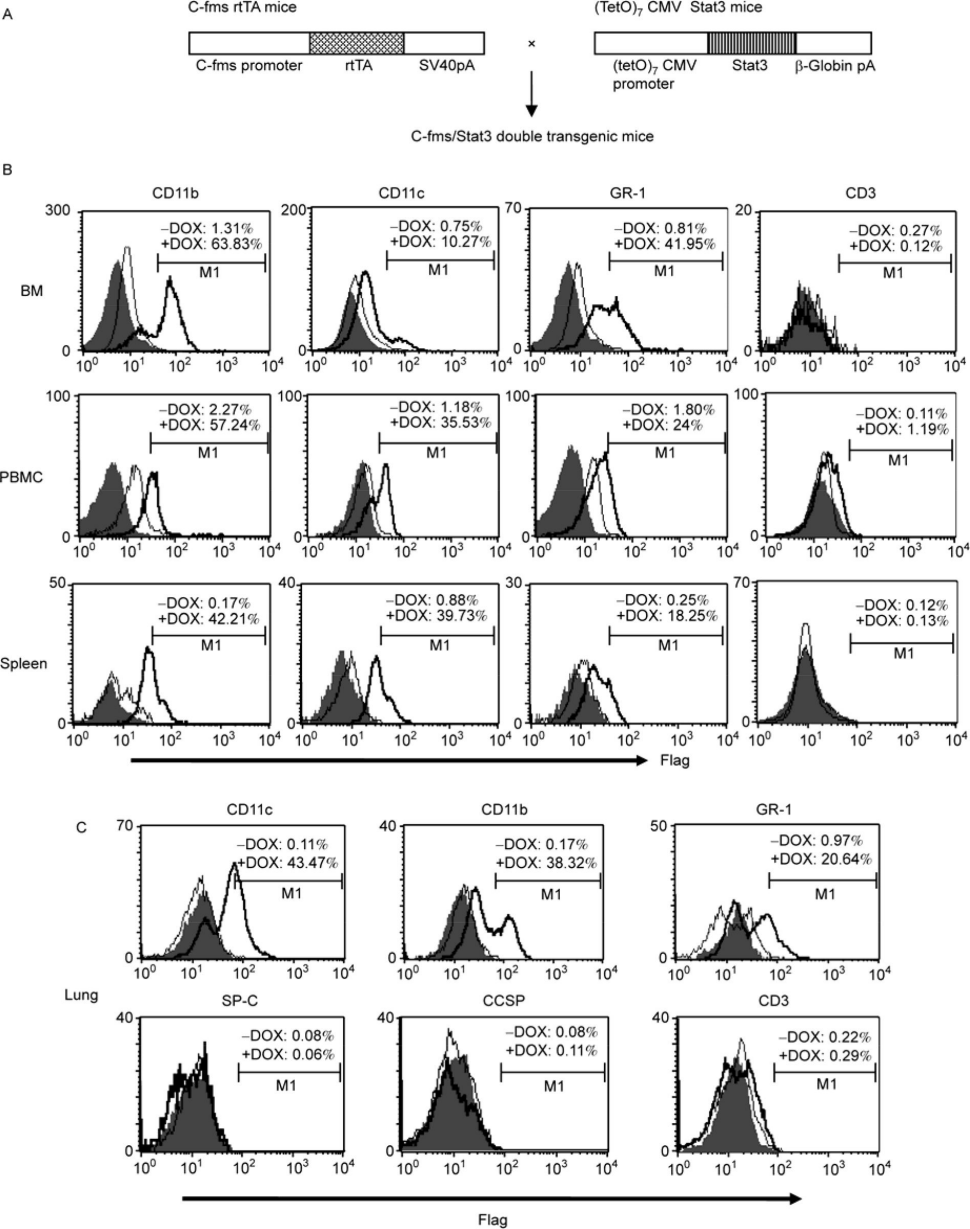
Generation of doxycycline-controlled c-fms-rtTA/(TetO)_7_-CMV-Stat3C double-transgenic mice. A, Construct maps for c-fms-rtTA single transgenic mice, (TetO)_7_-CMV-Stat3C single transgenic mice and double transgenic progeny. B, Myeloid specific expression of Stat3C-Flag fusion protein in c-fms-rtTA/(TetO)_7_-CMV-Stat3C double-transgenic mice. Cells from the bone marrow, blood (PBMC) and spleen of 3-month doxycycline-treated (+DOX, black line) and untreated (−DOX, gray line) bitransgenic mice were stained with anti-Flag antibody in combination with cell-surface markers CD11b, CD11c, GR-1 and CD3. In gated CD11b^+^, CD11c^+^, GR-1^+^ and CD3^+^ cells, Flag^+^ cells were analyzed by FACS in histograms. Isotype controls were shown as the shaded areas. C, The above study was repeated in the lung, except that cell-specific anti-SP-C (AT II epithelial cell marker) and −CCSP (Clara cell marker) antibodies were also used for analysis. M1 is a histogram marker excluding isotypic control (negative control).

To directly show that activation of the Stat3 pathway in myeloid-lineage cells can increase inflammation and BMSC-AT II cell conversion, a doxycycline-controlled bitransgenic mouse model was generated to specifically direct Stat3 expression in myeloid cells. Figure 2A illustrates myeloid lineage-specific expression of the c-fms-rtTA/(TetO)_7_-CMV-Stat3C bitransgenic gene. In this system, a previously established c-fms-rtTA transgenic mouse line [8] was crossbred with a previously generated (TetO)_7_-CMV-Stat3C transgenic mouse line [23]. In order to assess the expression pattern of Stat3C in c-fms-rtTA/(TetO)_7_-CMV-Stat3C bitransgenic mice, a Flag sequence was added at the C terminus of the Stat3C cDNA to distinguish exogenous Stat3C-Flag fusion protein from endogenous Stat3 protein. Bitransgenic mice were treated with or without doxycycline for 5 months and were sacrificed for FACS analysis. Single-cell suspensions from the bone marrow, blood, spleen and lung were double stained with Flag antibody and antibodies specific for macrophages (CD11b), dendritic cells (DCs, CD11c), neutrophils (GR-1), or T cells (CD3). Myeloid-lineage CD11b^+^ macrophages, Gr-1^+^ neutraphils and CD11c^+^ DCs all showed Stat3C-Flag overexpression in tested organs of doxycycline-treated bitransgenic mice. As a negative control, there was no Stat3C-Flag induction in CD3^+^ T lymphocytes, SP-C^+^ AT II epithelial cells and CCSP^+^ Clara cells regardless of doxycycline treatment in bitransgenic mice (Figure 2B and C). These results demonstrated that Stat3C-Flag fusion protein overexpression in c-fms-rtTA/(TetO)_7_-CMV-Stat3C bitransgenic mice was myeloid lineage-specific. No Stat3C-Flag fusion protein was detected in myeloid lineage cells in above mentioned organs of wild type mice regardless of doxycycline treatment (data not shown), suggesting that induction of Stat3C-Flag fusion protein was not caused by doxycycline alone.

### 2.3 Overexpression of Stat3C caused systemic inflammation and lung tissue neoplastic proliferation

**Figure 3:**
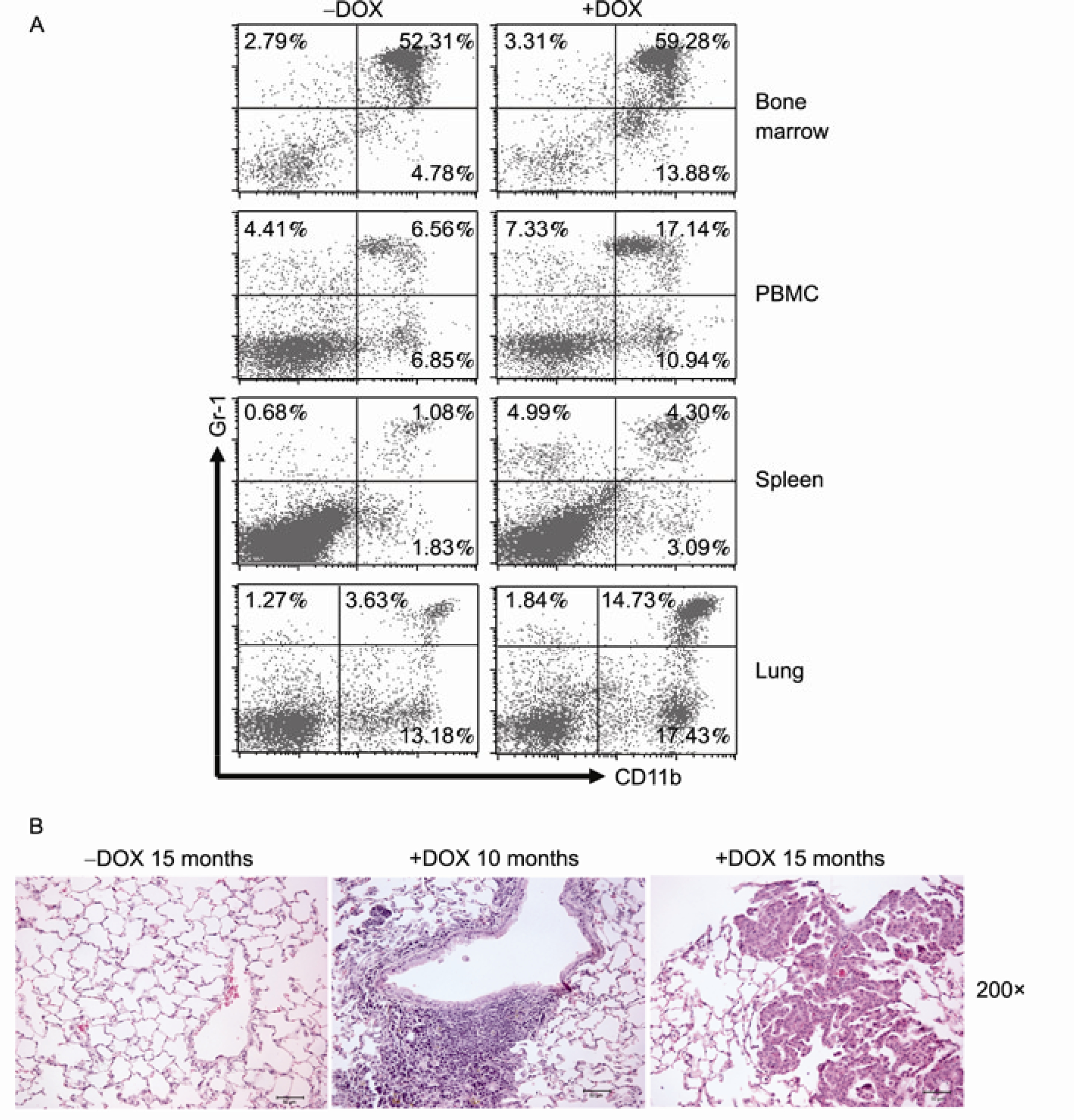
Systemic increases of myeloid cells in c-fms-rtTA/(TetO)_7_-CMV-Stat3C bitransgenic mice. A, Single cell suspensions were prepared from the bone marrow, blood (PBMC), spleen and lung of 3-month doxycycline-treated and untreated bitransgenic mice for FACS analyses. Distribution of CD11b^+^ macrophage and GR-1^+^ cells was presented in dot plots. Each experiment represents 3 independent (*n*=3) studies. –DOX, doxycycline-untreated bitransgenic mice; +DOX, doxycycline-treated bitransgenic mice. B, C-fms-rtTA/(TetO)_7_-CMV-Stat3 bitransgenic mice were treated or untreated with doxcycline for 10–15 months. Lungs were inflated, sectioned and analyzed by H&E staining.

C-fms-rtTA/(TetO)_7_-CMV-Stat3C bitransgenic mice were treated with or without doxycycline for 5 months. The cells from the bone marrow, blood, spleen and lung were isolated and stained with fluorochrome-conjugated antibodies specific for macrophages, DC, neutrophils, B cells or T cells for FACS analysis. Compared with those from doxycycline-untreated mice, the percentage of CD11b^+^ macrophages was increased from 6.85% to 10.94% in the blood (PBMC), 1.83% to 3.09% in the spleen and 13.18% to 17.43% in the lung of doxycycline-treated bitransgenic mice. The percentage of GR-1^+^ neutrophils was increased from 4.41% to 7.33% in the blood (PBMC), 0.68% to 4.99% in the spleen and remained unchanged in the lung of doxycycline-treated bitransgenic mice. Interestingly, the percentage of CD11b^+^ GR-1^+^ myeloid cells was increased from 6.56% to 17.14% in the blood, 1.08% to 4.30% in the spleen and 3.63% to 14.73% in the lung of doxycycline-treated bitransgenic mice (Figure 3A). The absolute numbers of these cells were also systematically increased (Table 1). Therefore, Stat3C overexpression in myeloid lineage cells can increase systemic myeloid cells (especially CD11b^+^ GR-1^+^ cells) in various organs of bitransgenic mice. In the lung, pathogenesis of doxycycline-treated or untreated c-fmsr-tTA/(TetO)_7_-CMV-Stat3C bitransgenic mice was further analyzed by histology. Lung inflammation and neoplastic pneumocyte proliferation were observed in 10–15 month doxycycline-treated bitransgenic mice (Figure 3B). In contrast to myeloid cells, changes of T and B lymphocytes were not observed in bitransgenic mice regardless of doxycycline treatment (Table 1).

### 2.4 BMSC markers

To assess BMSC-AT II cell conversion, a previously published method was used to obtain BMSCs that were enriched from the whole bone marrow via plastic adherent of fibroblastoid cell fraction in specific culture medium [7]. Although this fractionation method was used for isolation of BMSCs, little is known about markers that define self-renewal BMSCs. In our cultured condition, BMSCs were mouse hematopoietic lineage panel negative (including biotin-conjugated anti-mouse CD3ε, CD11b (Mac-1), CD45R/B220, TER-119, Ly-6G and Ly-6C) (data not shown). From 0 to 19 passages, BMSCs expressed relatively high levels of Scal-1, SSEA-1, CD44, CD106 and apoptosis inhibitor 6 (Api6/AIM/Spα) under the cultured condition (Table 2). CD106, CD44 and SSEA-1 were previously defined markers for BMSCs [30–32]. Expression of Api6 was identified for the first time in cultured BMSCs. By contrast, embryonic stem cell markers, such as OCT-4, BMP4 and SOX2, and Nanog, were expressed at relatively low levels under the same cultured condition (Table 2), even though Nanog and Sox2 showed increased expression in BMSCs at later culturing passages (11–19). This suggests that BMSCs and embryonic stem cells have different characteristics of marker expression, implicating differential function properties *in vivo*. In addition, cultured BMSCs did not express AT II epithelial marker SP-C throughout culture passages. In the subsequent studies, BMSCs with 9–13 passages were routinely used for injection studies.

### 2.5 Repopulation of donor BMSCs in c-fms-rtTA/(TetO)_7_-CMV-Stat3C bitransgenic mice

**Figure 4:**
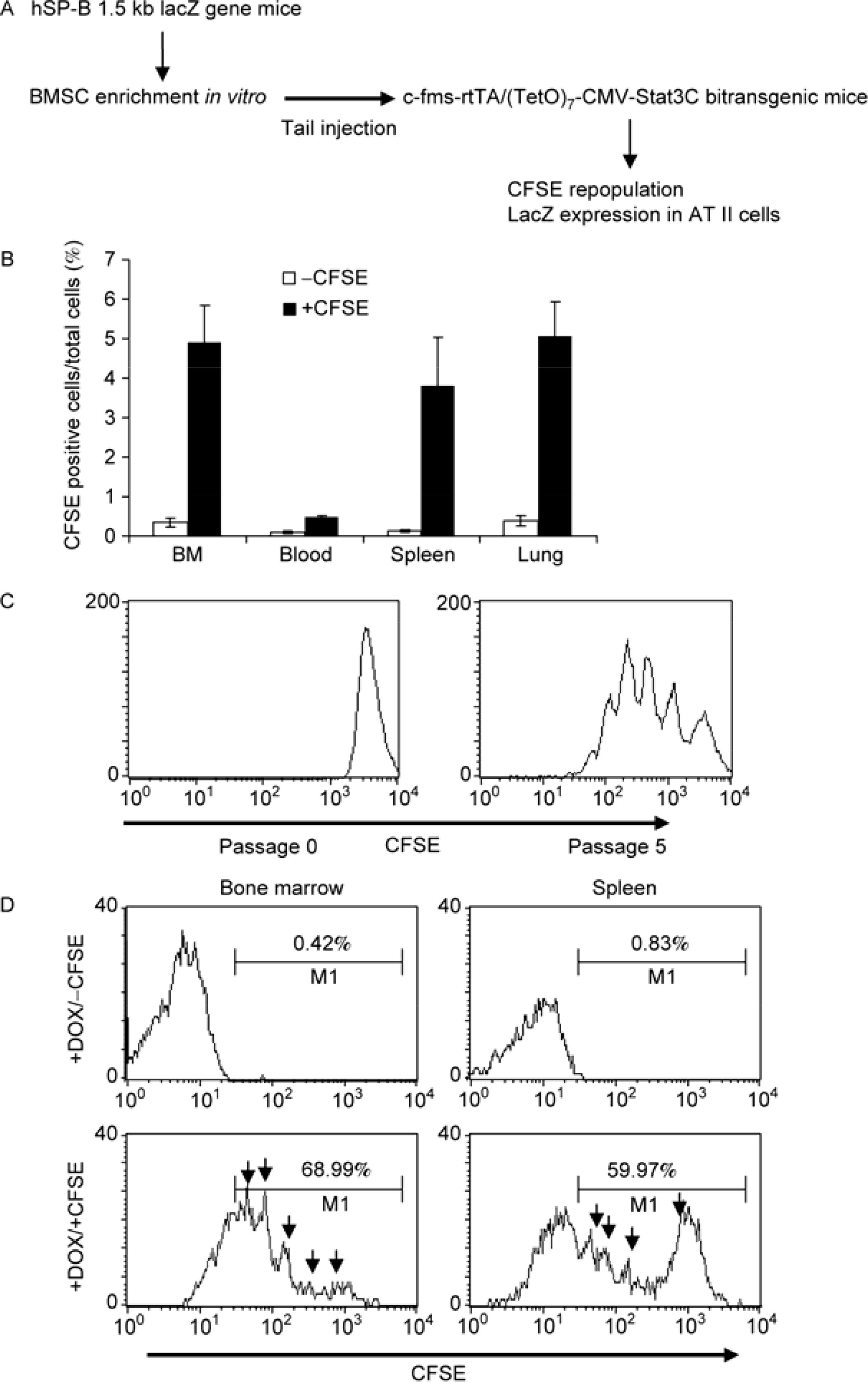
Repopulation of hSP-B 1.5-kb lacZ adherent BMSCs in c-fms-rtTA/(TetO)_7_-CMV-Stat3C bitransgenic mice by CFSE analysis. A, Schematic illustration of BMSC transplantation experiment. B, CFSE-labeled hsp-B 1.5-kb lacZ adherent BMSCs after 9 passages were injected into doxycycline-treated c-fms-rtTA/(TetO)_7_-CMV-Stat3C bitransgenic mice. After 2 weeks, distribution of donor BMSCs was detected by flow cytometry in various organs of the recipient c-fms-rtTA/(TetO)_7_-CMV-Stat3C bitransgenic mice. Non-CFSE-labeled hsp-B 1.5-kb lacZ adherent BMSCs were used as control. C, Cell division of CFSE-labeled hSP-B 1.5-kb lacZ adherent BMSCs (+CFSE) was detected in the cultured condition after 5 passages, but not in 0 passage. D, Cell division of CFSE-labeled hSP-B 1.5-kb lacZ adherent BMSCs (+CFSE) was detected in the bone marrow and spleen of recipient doxycycline-treated c-fms-rtTA/(TetO)_7_-CMV-Stat3C bitransgenic mice. Cell cycles of CFSE-labeled adherent BMSCs are indicated by arrows (*n*=3). M1 is a histogram marker excluding isotypic controls (negative controls). No BMSCs proliferation was observed in doxycycline-untreated recipient c-fms-rtTA/(TetO)_7_-CMV-Stat3C bitransgenic mice (Figure S1).

The experimental design for the bone marrow transplantation study involving c-fms-rtTA/(TetO)_7_-CMV-Stat3C bitransgenic mice and hSP-B 1.5-kb lacZ transgenic mice is illustrated in Figure 4A. First, organ repopulation and self-renew of injected adherent BMSCs in c-fms-rtTA/(TetO)_7_-CMV-Stat3C bitransgenic mice was assessed by carboxyfluoresein diacetate succinimidyl ester (CFSE) in cell tracing experiment. After enrichment by 9 passages in *in vitro* culturing, adherent BMSCs from hSP-B 1.5-kb lacZ transgenic mice were further purified by CD11b, CD34 and CD45 magnetic beads to remove macrophages and endothelial cells. Purified BMSCs were labeled by CFSE and tail vein injected into doxycycline-treated or untreated c-fms-rtTA/(TetO)_7_-CMV-Stat3C bitransgenic mice. Two weeks later, single-cell suspensions were prepared from the bone marrow, spleen and lung of recipient mice. As demonstrated in Figure 4B, CFSE-labeled cells were readily detected in these organs of doxycycline-treated bitransgenic mice, but not in untreated bitransgenic mice (data not shown). This indicates that migration and homing of injected adherent BMSCs in recipient bitransgenic mice is associated with inflammation caused by Stat3 overexpression in myeloid lineage cells. Enriched BMSCs were able to proliferate as demonstrated in the cultured condition by a cell replication (proliferation) study, in which divided daughter cells (represented by different peaks) were separated by CFSE labeling (Figure 4C). After injection, adherent BMSCs were able to replicate in the bone marrow and spleen of doxycycline-treated c-fms-rtTA/(TetO)_7_-CMV-Stat3C bitransgenic mice by CFSE labeling (Figure 4D). No cell division of CFSE-labeled adherent BMSCs was observed in doxycycline-untreated recipient c-fms-rtTA/(TetO)_7_-CMV-Stat3C bitransgenic mice (Figure S1). Therefore, donor adherent BMSCs were able to self-renew primarily in the bone marrow and spleen of doxycycline-treated c-fms-rtTA/(TetO)_7_-CMV-Stat3C bitransgenic mice.

### 2.6 Conversion of hSP-B lacZ 1.5-kb BMSCs into AT II epithelial cells in c-fms-rtTA/(TetO)_7_-CMV-Stat3C bitransgenic mice

**Figure 5:**
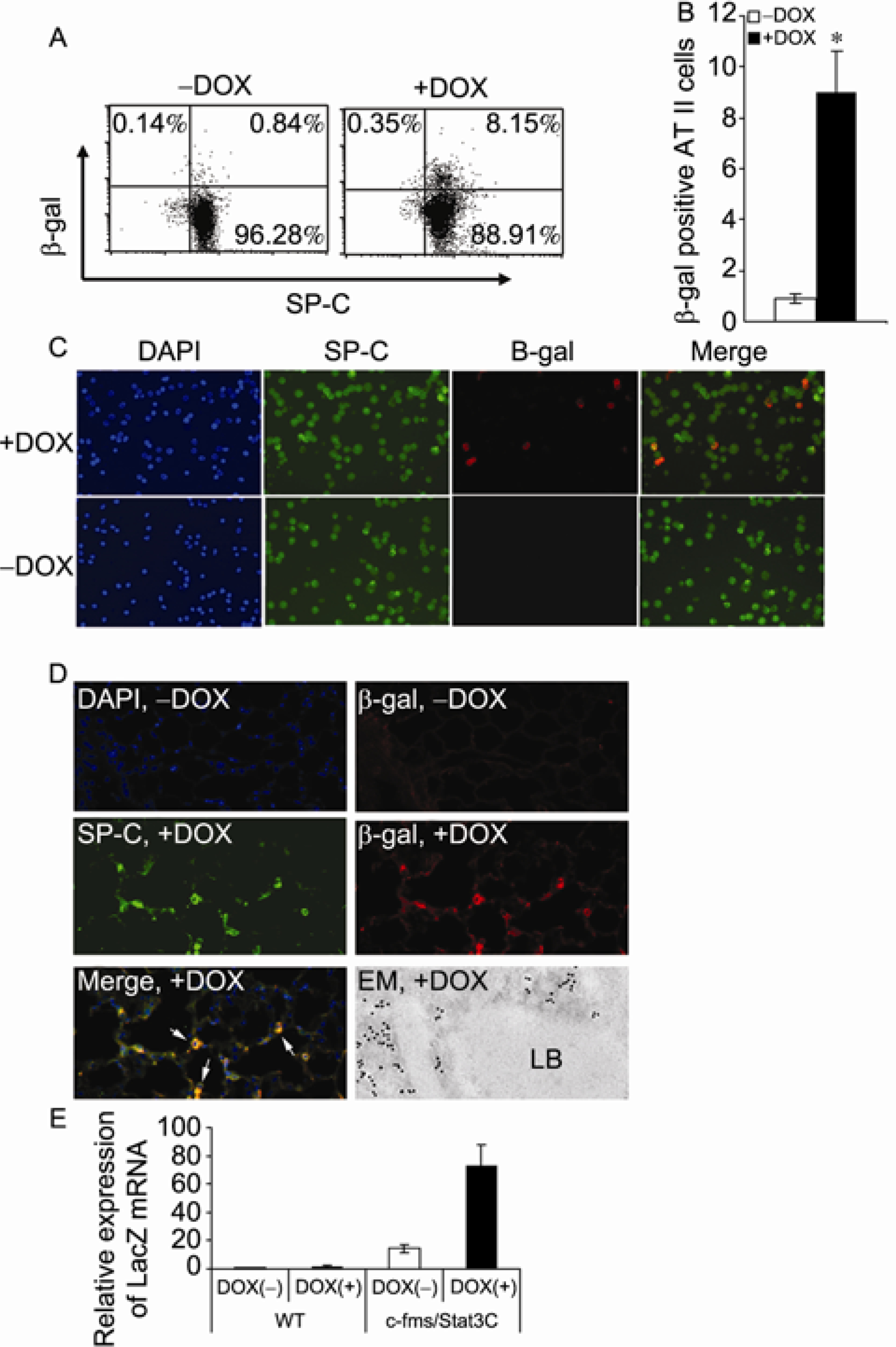
Conversion of hSP-B 1.5 kb lacZ adherent BMSCs into AT II epithelial cells in recipient c-fms-rtTA/(TetO)_7_-CMV-Stat3C bitransgenic mice. CD34- and CD45-negatively selected 9th passaged hSP-B 1.5-kb lacZ BMSCs were injected into c-fms-rtTA/(TetO)_7_-CMV-Stat3C bitransgenic mice with (+Dox) or without (−Dox) doxycycline treatment. AT II epithelial cells were isolated from recipient mice for protein labeling and mRNA analyses. A, A representative of FACS analysis by anti-SP-C and β-galactosidase antibodies. B, The percentage numbers of proSP-C/β-galactosidase double positive cells from 4 independent FACS studies (*n*=4). *, *P*<0.01. C, Colocalization of proSP-C and β-galactosidase double positive cells by immunofluorescence staining in isolated AT II epithelial cells. No colocalization was observed in doxycycline-untreated mice. Blue, DAPI staining of cell nuclei; green, pro-SP-C staining; red, β-galactosidase staining; orange, double positive staining. D, Colocalization of proSP-C and β-galactosidase double positive cells by immunofluorescence staining on lung tissue sections. Double positive cells are pointed out by arrows (orange). No colocalization was observed in doxycycline-untreated mice. Green, pro-SP-C staining; red, β-galactosidase staining. DAPI staining represents nuclei in the lung. E, Expression of lacZ mRNA in AT II epithelial cells of bitransgenic mice (*n*=3). Wild type mice were used as negative control.

To test if hSP-B 1.5-kb lacZ adherent BMSCs were able to convert into AT II epithelial cells in the lung of c-fms-rtTA/(TetO)_7_-CMV-Stat3C bitransgenic mice under the inflammatory condition, cultured (9 passages) and purified hSP-B 1.5-kb lacZ adherent BMSCs were injected into doxycycline-treated and untreated c-fms-rtTA/(TetO)_7_-CMV-Stat3C bitransgenic recipient mice. Six weeks after injection, AT II epithelial cells were isolated from recipient mice and stained with rabbit anti-proSP-C antibody and mouse β-galactosidase antibody for flow cytometry (Figure 5A). Approximate 8%–9% of proSP-C-positive cells showed β-galactosidase co-staining in doxycycline-treated bitransgenic mice (Figure 5B). This observation was confirmed by double immunofluorescence staining at a conversion rate of 10% in doxycycline-treated mice, but not in untreated mice (Figure 5C). No co-staining was observed in the doxycycline-untreated bitransgenic lung. To prove that these converted cells were in indeed located within the alveolar epithelium of the alveolar walls, double immunofluorescence staining was performed on tissue sections (Figure 5D). SP-C and β-galactosidase signals were overlapped in some AT II epithelial cells located in the corner of the alveolar walls. In a systematical study, hSP-B 1.5-kb lacZ adherent BMSCs cultured *in vitro* at various passage time points were injected into c-fms-rtTA/(TetO)_7_-CMV-Stat3C bitransgenic mice to assess the ability of BMSC conversion into AT II epithelial cells. After 11 and 13 passages, hSP-B 1.5-kb lacZ adherent BMSCs were still able to convert into AT II epithelial cells in doxycycline-treated c-fms-rtTA/(TetO)_7_-CMV-Stat3C bitransgenic mice using the same methods as outlined above. However, after 19 passages *in vitro*, hSP-B 1.5-kb lacZ adherent BMSCs failed to convert into AT II epithelial cells in c-fms-rtTA/(TetO)_7_-CMV-Stat3C bitransgenic mice regardless of doxycycline treatment (data not shown).

Increased expression of β-galactosidase was partially due to transcriptional up-regulation since the Real-Time PCR analysis showed increased mRNA expression in AT II epithelial cells that were isolated from the lungs of recipient c-fms-rtTA/(TetO)_7_-CMV-Stat3C bitransgenic mice (Figure 5E). It is notable that there was a low level expression of lacZ mRNA in doxycycline-untreated mice, indicating a minor leakage in the system. This was also observed in Flag expression (PBMC; Figure 2). As a control, no β-galactosidase mRNA expression was detected in wild type recipient mice regardless of doxycycline treatment after injection of hSP-B 1.5-kb lacZ adherent BMSCs (Figure 5E).

### 2.7 Bone marrow transplantation

**Figure 6:**
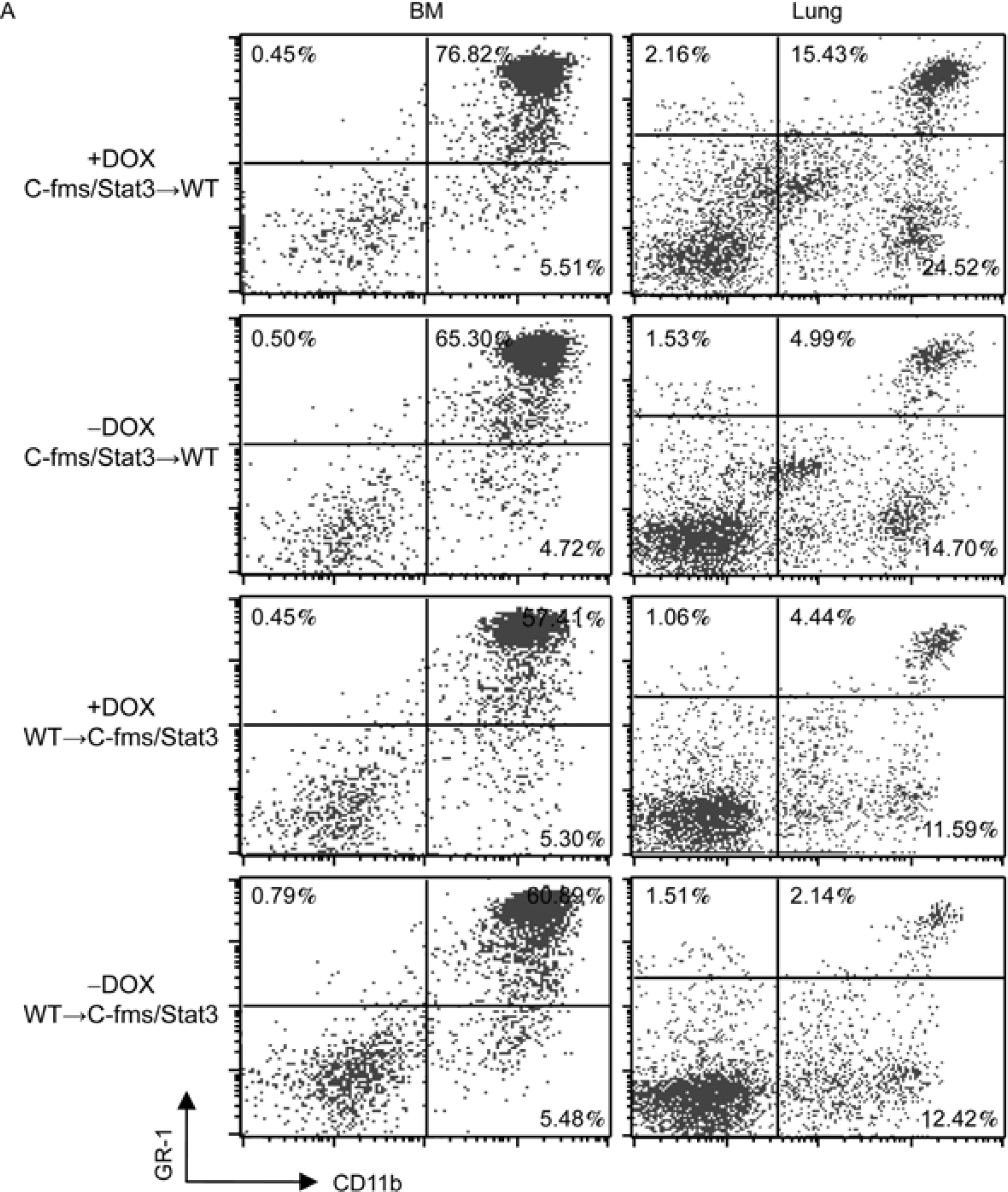

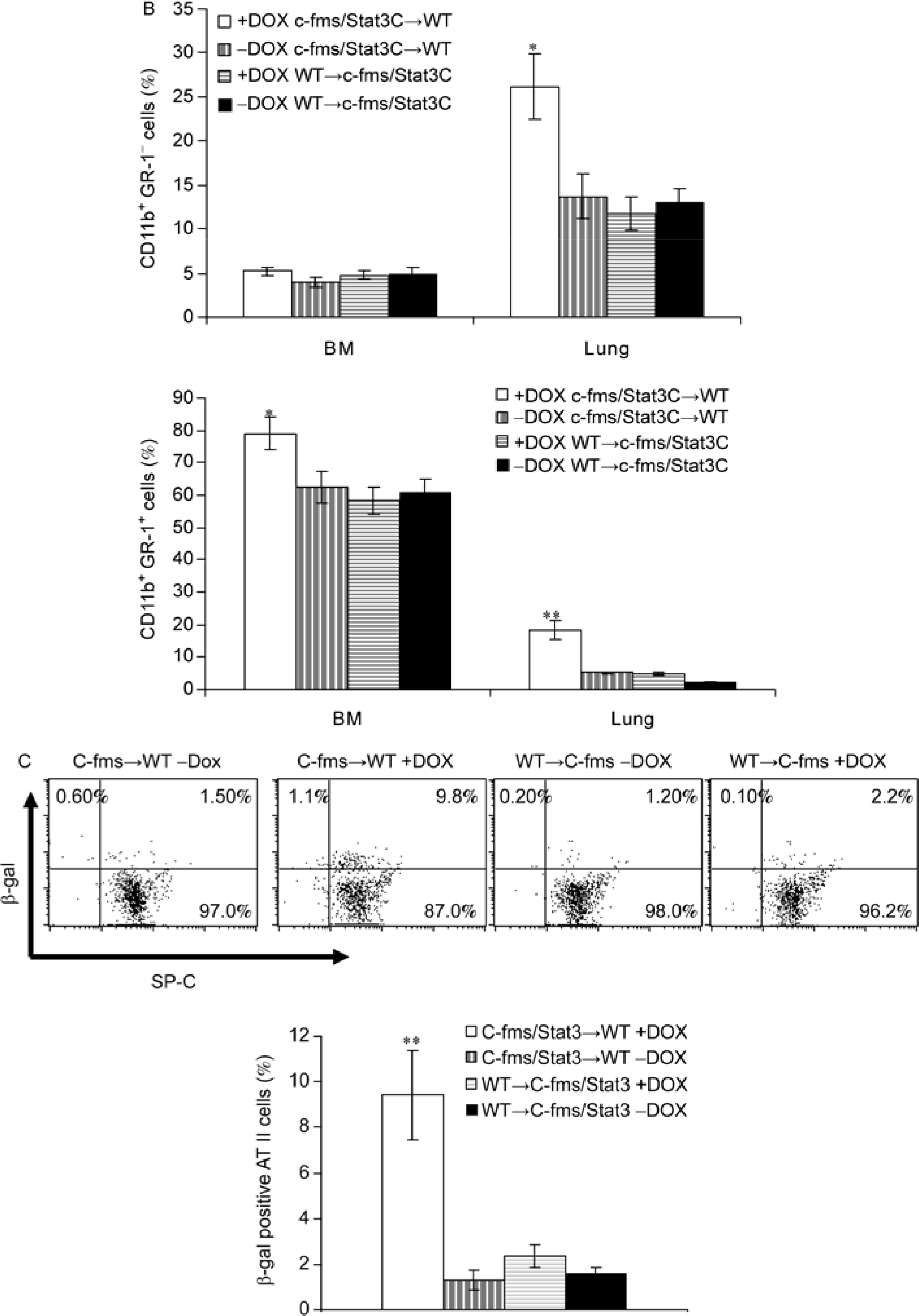
Inflammation and BMSC analyses in chimeric mice. Bone marrow cells from wild-type or c-fms-rtTA/(TetO)_7_-CMV-Stat3C bitransgenic mice (CD45.1^+^) were transplanted into reciprocal mice (CD45.2^+^) that were lethally irradiated. Chimeric mice were treated with or without doxycycline. A, A representative analysis of CD11b^+^ and GR-1^+^ cells in the bone marrow and lung of chimeric mice by FACS. B, The percentage numbers of CD11b^+^ GR-1^+^ and CD11b^+^ GR-1^−^ in the bone marrow and lung were analyzed in three independent studies (*n*=3–4). *, *P*<0.05; **, *P*<0.01. C, Two months after bone marrow transplantation and doxycycline treatment, cultured adherent 9th passage BMSCs were injected into chimeric mice. Six weeks later, AT II epithelial cells were isolated from recipient mice for FACS analysis by anti-SP-C and β-galactosidase antibodies. A representative analysis and the percentage of proSP-C/β-galactosidase double positive cells from 4 independent FACS (*n*=3–4) are demonstrated. **, *P*<0.01.

Although the above studies demonstrated that myeloid Stat3C-induced inflammation caused BMSCs-AT II cell conversion, they do not address a question whether these phenomenon is due to malfunction of myeloid lineage progenitor cells, or due to the microenvironment change that affects progenitor cell development as a result of Stat3C overexpression in recipient mice. To address this issue, bone marrow cells from wild-type or c-fms-rtTA/(TetO)_7_-CMV-Stat3C bitransgenic mice (both CD45.1^+^) were transplanted into reciprocal recipients (CD45.2^+^) that were lethally irradiated to generate bone marrow chimeric mice. After three months, recipient mice were infused with *in vitro* cultured (9 passages) and purified hSP-B 1.5-kb lacZ adherent BMSCs through tail vein of doxycycline-treated or untreated recipient mice. After 6 weeks of BMSCs injection, the bone marrow, blood, spleen and lung were harvested from various groups for analysis. Recipient cells were excluded by gating on CD45.1^+^ cells that were derived from the donor mice. Four experimental bone marrow transplantation (BMT) groups were designed including doxycycline-untreated (−DOX, c-fms/Stat3C→WT; −DOX WT→WT) or treated (+DOX, c-fms/Stat3C→WT; +DOX, WT→WT) samples. First, myeloid lineage cells were analyzed (Figure 6A and B). The percentage of CD11b^+^ GR-1^+^ cells in the bone marrow (65.30%–76.82%) and lung (4.99%–15.43%) of the doxycycline-treated c-fms/Stat3C→WT BMT group was increased compared with those of the doxycycline-untreated c-fms/Stat3C→WT BMT group. The percentage of CD11b^+^ macrophages in the lung of the same group was increased from 14.70% to 24.52% and remained relatively unchanged in the bone marrow. The percentage of GR-1^+^ neutrophils in the lung of the same group was modestly increased from 1.53% to 2.16% and remained relatively unchanged in the bone marrow. In the control WT→WT BMT group, CD11b^+^GR-1^+^, CD11b^+^ and GR-1^+^ cells in the doxycycline-treated group were not significantly different from those in the doxycycline-untreated group (data not shown). In association with these observations, proSP-C and β-galactosidase double positive AT II epithelial cells were increased to 9.8% by flow cytometry in the doxycycline-treated c-fms/Stat3C→WT BMT group, but not in other groups (Figure 6C). Therefore, Stat3C-induced autonomous of myeloid progenitor cells was the cause of BMSC-AT II cell conversion in c-fms-rtTA/(TetO)_7_-CMV-Stat3C bitransgenic mice.

To assess contribution made by the microenvironmental change in c-fms-rtTA/(TetO)_7_-CMV-Stat3C bitransgenic mice, additional BMT groups were designed including doxycycline-treated or untreated WT→c-fms/Stat3C samples. As demonstrated in Figure 6A and B, no distinct changes of CD11b^+^GR-1^+^, CD11b^+^ and GR-1^+^ cells were observed in reciprocal transplanted mice. As a consequence, minimal BMSC-AT II cell conversion was observed (Figure 6C). This result excluded the animal microenvironment as a cause to alter development of myeloid lineage progenitor cells and BMSC-AT II cell conversion.

## 3 Discussion

As we reported previously, Stat3 is a pro-inflammatory and oncogenic molecule. Over-activation of the Stat3 pathway mobilizes inflammatory cells to influx into the lung [18]. In this report, FACS analysis showed that Stat3 was activated in highly proliferative CD11b^+^ and GR-1^+^ cells in multiple organs of *lal*^−/−^ mice (Figure 1). It is possible that Stat3 activation in myeloid cells mediates inflammation and BMSC conversion to AT II epithelial cells as observed in *lal*^−/−^ mice [7]. To prove this assumption, a myeloid-specific doxycycline-inducible c-fms-rtTA/(TetO)_7_-CMV-Stat3C bitransgenic model system was established. FACS analysis with Flag antibody and cell specific antibodies showed myeloid cell-specific expression of Stat3C-Flag fusion protein in the bone marrow, blood, spleen and lung (Figure 2B). This is in agreement with our previous findings [8,11,12,33]. As a consequence, Stat3C over-expression in myeloid cells caused systemic increase of macrophages and neutraphils in bitransgenic mice. Importantly, the number of CD11b^+^GR-1^+^ positive MDSCs was significantly increased (Figure 3A). MDSCs have great potential to facilitate the processes of neoplastic tissue remodeling [34–36]. Indeed, hyperplasia was observed in doxycycline-treated c-fms-rtTA/(TetO)_7_-CMV-Stat3C bitransgenic lungs (Figure 3B).

In association with inflammation, BMSCs were analyzed to see if activation of the Stat3 pathway induces BMSC migration, repopulation and conversion into AT II epithelial cells in this animal model. To have a better understanding of BMSCs in our *in vitro* cultured condition, multiple markers were analyzed to define BMSC at multi-passage points. BMSCs cultured in our condition were low on expression of embryonic stem cell markers (OCT-4, BMP4, SOX2, and Nanog), and high on expression of Scal-1, SSEA-1, CD44 and CD106 markers. Sca-1 expression correlates with the ability of murine BM stromal cells to support proliferation of BM cells [37]. SSEA-1 defined mesenchymal compartment has a high capacity to differentiate to astrocyte-, endothelial- and hepatocyte-like cells *in vitro* [32]. CD44 and CD106 are adherent molecules. Modification of the glycans on CD44 increases the efficiency of BMSCs delivery and homing to the bone marrow [31]. CD106 expression has been linked to the hematopoietic supportive capacity of immortalized murine BM stromal cell lines [38,39]. In our analysis, expression of Api6 was also high in cultured BMSCs. Api6 is a secreted protein and belongs to the macrophage scavenger receptor cysteine-rich domain superfamily (SRCR-SF) [40,41]. Although Api6 was originally identified from macrophages, its expression was also identified in AT II epithelial cells of *lal*^−/−^ mice [29]. Over-expression of this molecule in myeloid cells and lung AT II epithelial cells using the doxycycline-inducible systems stimulated proliferation and influx of macrophages, neutrophils and MDSCs *in vivo*, which resulted in lung adenocarcinoma [33,42]. Since expression of these markers were varied in different passages, BMSCs from different passages were injected into c-fms-rtTA/(TetO)_7_-CMV-Stat3C bitransgenic mice to verify plasticity.

Similar to *lal*^−/−^ mice, hSP-B 1.5-kb lacZ adherent BMSCs were able to migrate into the bone marrow, spleen and other organs such as the lung (Figure 4B). BMSCs were only able to self-renew in the bone marrow and spleen, not in the blood and lung as analyzed by the CFSE tracing study (Figures 4D and S1). Once being recruited into the lung, adherent BMSCs showed the ability to convert into AT II epithelial cells by Real-Time PCR, flow cytometry, and double immunofluorescence staining (Figure 5). Since this conversion did not occur in doxycycline-untreated bitransgenic mice, inflammation triggered by Stat3C overexpression in myeloid cells is a determining factor for BMSC recruitment and conversion. This observation is consistent with our previous finding in *lal*^−/−^ mice that adherent subpopulation of bone marrow cells from hSP-B 1.5-kb lacZ donor mice can convert into AT II epithelial cells under the inflammatory and tissue remodeling condition [7]. While hSP-B 1.5-kb lacZ adherent BMSCs after 9, 11 and 13 passages from *in vitro* culturing were able to convert into AT II epithelial cells, the same cell population after 20 passages lost the ability of conversion in c-fms-rtTA/(TetO)_7_-CMV-Stat3C bitransgenic mice.

To clarify whether systemic inflammation and BMSC conversion into AT II epithelial cells are due to malfunction of myeloid progenitor cells in the bone marrow, or due to the microenvironment change in bitransgenic mice after Stat3C over-expression, whole bone marrow transplantation was performed to make chimeric mice. After whole bone marrow transplantation for three months, recipient wild type mice that were received bone marrow cells from c-fms-rtTA/(TetO)_7_-CMV-Stat3C bitransgenic mice developed systemic inflammation and showed BMSCs-AT II cell conversion with doxycycline treatment. In a reverse bone marrow transplantation, inflammation and BMSCs-AT II cell conversion disappeared in c-fms-rtTA/(TetO)_7_-CMV-Stat3C bitransgenic mice after receiving bone marrow cells from wild type mice regardless of doxycycline treatment (Figure 6). These results clearly demonstrated that alteration of bone marrow progenitor cells, rather than the microenvironment change, contributes to systematic inflammation and BMSCs-AT II cell conversion in c-fms-rtTA/(TetO)_7_-CMV-Stat3C bitransgenic mice.

As proposed for BMSCs to repair the injured lung [43], several conditions must be met: (i) BMSCs must be available in the bone marrow; (ii) BMSCs must be mobilized from the bone marrow and traffic to the site of injury; (iii) BMSCs must be integrated into the existing lung structure, divide and differentiate. Based on our observations, these events are highly associated with inflammation and myeloid cell influx into the lung, suggesting that some common mechanisms regulate behaviors of both myeloid cells and BMSCs. Since both cells are originated in the bone marrow, myeloid cells exert a great impact on BMSC functions. Myeloid cells synthesize and secret many cytokines and chemokines, which not only regulate the myeloid cell behavior and function, but also influence BMSCs in a paracrine fashion in the bone marrow, circulating system and tissue organs. In this report, we demonstrated that Stat3 plays a critical role in myeloid cell proliferation and function. As a potent transcription factor, Stat3 induces expression of many pro-inflammatory cytokines and chemokines *in vivo* as we previously demonstrated [18]. These molecules facilitate recruitment of myeloid cells into the damaged organs. It is highly possible that the same group of cytokines and chemokines mobilizes BMSCs to the damaged site. Identification of these molecules will greatly benefit regenerative medicine and cell-based gene therapy. Since both MDSCs and BMSCs suppress T cell proliferation/function to subvert immune surveillance and prevent the immune system from eliminating tumor cells [34–36,44,45], whether bone marrow-derived BMSCs participate in organ pathogenic development along with MDSCs in *lal*^−/−^ mice and c-fms-rtTA/(TetO)_7_-CMV-Stat3C bitransgenic mice is an intriguing idea and remains to be tested in the future.

## Supplementary Material

Supplementary material **Figure S1** The control study duplicating Figure 4D, except that doxycycline-untreated c-fms-rtTA/(TetO)7-CMV-Stat3C bitransgenic mice were used. Cell division of CFSE-labeled hSP-B 1.5-kb lacZ adherent BMSCs (+CFSE) was not detected in the bone marrow and spleen of recipient mice.

## Figures and Tables

**Table 1:** The absolute numbers of inflammatory cells in c-fms-rtTA/(TetO)_7_-CMV-Stat3C bitransgenic mice^a)^

Cell types	Bone marrow (×10^6^)		Spleen (×10^6^)		Lung (×10^5^)	
	−DOX	+DOX	−DOX	+DOX	−DOX	+DOX
CD11b+GR-1+	28.12±3.35	37.24±5.87*	0.46±0.05	3.12±1.05*	0.41±0.11	1.58±0.26*
CD11b+GR-1−	2.77±1.02	7.62±2.46*	1.38±0.34	2.34±0.96	1.4±0.36	1.72±0.09
CD11b−GR-1+	1.53±0.66	2.05±0.86	1.05±0.23	3.33±0.19*	0.16±0.02	0.39±0.08*
CD4+	0.69±0.22	0.68±0.36	13.71±3.07	11.93±2.06	0.14±0.02	0.11±0.03
CD8+	0.48±0.07	0.46±0.07	6.31±1.11	5.53±0.42	0.09±0.01	0.08±0.02
B220+	15.55±4.93	14.66±4.08	40.8±6.27	44.1±7.31	0.17±0.03	0.18±0.04

a) * Significant increase, *P*<0.05.

**Table 2:** Expression of markers in *in vitro* cultured BMSCs^a)^

Passages	0th (%)	5th (%)	7th (%)	9th (%)	11th(%)	13th (%)	15th (%)	17th (%)	19th (%)
BMSC marker									
Sca-1	2.14	44.92	56.93	65.73	72.12	78.66	77.91	78.61	81.94
SSEA-1	6.36	23.47	24.44	26.23	16.3	10.7	5.42	4.31	3.27
Api6	2.16	8.84	11.97	43.78	14.6	20.31	32.78	33.17	29.89
CD44	93.29	82.65	70.97	79.56	94.76	83.32	99.42	89.02	98.96
CD106	6.15	81.55	80.1	83.21	57.61	50.71	57.69	59.61	49.25
Embryonic stem cell marker									
OCT-3/4	1.89	1.07	1.04	1.2	1.47	1.7	1.39	3.17	4.29
Nanog	0.78	1.97	3.61	1.8	5.23	8.08	12.42	12.9	19.2
BMP4	0.34	0.86	1.29	1.41	1.94	1.23	3.74	1.92	3.23
SOX2	6.10	1.04	1.35	1.54	4.68	5.28	10.06	9.72	8.03
Type II epithelial marker									
SP-C	0.71	1.02	1.6	0.51	0.22	0.14	0.09	0.04	0.03

a) Numbers represent percentage (%) of positive cells that express markers. BMSC, bone marrow stem cells; SSEA-1, stage-specific embryonic antigen-1; Api6, apoptosis inhibitor 6; OCT-3/4, octamer-3/4; BMP4, bone morphogenic protein-4; SOX2, sex determining region Y-Box 2; SP-C, surfactant protein C.

## References

[1] Whitsett JA, Weaver TE. Hydrophobic surfactant proteins in lung function and disease. N Engl J Med, 2002, 347: 2141–2148 12501227 10.1056/NEJMra022387

[2] Yan C, Du H. Alveolus formation: what have we learned from genetic studies? J Appl Physiol, 2004, 97: 1543–1548 15358757 10.1152/japplphysiol.00286.2004

[3] Bishop AE Pulmonary epithelial stem cells. Cell Prolif, 2004, 37: 89–96 14871239 10.1111/j.1365-2184.2004.00302.x PMC6495778

[4] Williams MC Alveolar type I cells: molecular phenotype and development. Annu Rev Physiol, 2003, 65: 669–695 12428023 10.1146/annurev.physiol.65.092101.142446

[5] Mason RJ, Williams MC, Moses HL, Stem cells in lung development, disease, and therapy. Am J Respir Cell Mol Biol, 1997, 16: 355–363 9115744 10.1165/ajrcmb.16.4.9115744

[6] Weiss DJ, Kolls JK, Ortiz LA, Stem cells and cell therapies in lung biology and lung diseases. Proc Am Thorac Soc, 2008, 5: 637–667 18625757 10.1513/pats.200804-037DW PMC2645238

[7] Yan C, Lian X, Dai Y, Gene delivery by the hSP-B promoter to lung alveolar type II epithelial cells in LAL-knockout mice through bone marrow mesenchymal stem cells. Gene Ther, 2007, 14: 1461–1470 17700706 10.1038/sj.gt.3303006

[8] Yan C, Lian X, Li Y, Macrophage-specific expression of human lysosomal acid lipase corrects inflammation and pathogenic phenotypes in *lal*^−/−^ mice. Am J Pathol, 2006, 169: 916–926 16936266 10.2353/ajpath.2006.051327 PMC1698822

[9] Qu P, Yan C, Blum JS, Myeloid-specific expression of human lysosomal acid lipase corrects malformation and malfunction of myeloid-derived suppressor cells in *lal*^−/−^ mice. J Immunol, 2011, 187: 3854–3866 21900179 10.4049/jimmunol.1003358 PMC3178672

[10] Qu P, Du H, Li Y, Myeloid-specific expression of Api6/AIM/Sp alpha induces systemic inflammation and adenocarcinoma in the lung. J Immunol, 2009, 182: 1648–1659 19155514 10.4049/jimmunol.182.3.1648 PMC2630116

[11] Qu P, Yan C, Du H. Matrix metalloproteinase 12 overexpression in myeloid lineage cells plays a key role in modulating myelopoiesis, immune suppression, and lung tumorigenesis. Blood, 2011, 117: 4476–4489 21378275 10.1182/blood-2010-07-298380 PMC3099569

[12] Wu L, Yan C, Czader M, Inhibition of PPARgamma in myeloid-lineage cells induces systemic inflammation, immunosuppression, and tumorigenesis. Blood, 2012, 119: 115–126 22053106 10.1182/blood-2011-06-363093 PMC3251224

[13] Akira S, Nishio Y, Inoue M, Molecular cloning of APRF, a novel IFN-stimulated gene factor 3 p91- related transcription factor involved in the gp130-mediated signaling pathway. Cell, 1994, 77: 63–71 7512451 10.1016/0092-8674(94)90235-6

[14] Wegenka UM, Lutticken C, Buschmann J, The interleukin-6-activated acute-phase response factor is antigenically and functionally related to members of the signal transducer and activator of transcription (STAT) family. Mol Cell Biol, 1994, 14: 3186–3196 8164674 10.1128/mcb.14.5.3186-3196.1994 PMC358686

[15] Zhong Z, Wen Z, Darnell JE Jr. Stat3: a STAT family member activated by tyrosine phosphorylation in response to epidermal growth factor and interleukin-6. Science, 1994, 264: 95–98 8140422 10.1126/science.8140422

[16] Leonard WJ, O’Shea JJ. Jaks and STATs: biological implications. Annu Rev Immunol, 1998, 16: 293–322 9597132 10.1146/annurev.immunol.16.1.293

[17] Taga T, Kishimoto T. Gp130 and the interleukin-6 family of cytokines. Annu Rev Immunol, 1997, 15: 797–819 9143707 10.1146/annurev.immunol.15.1.797

[18] Li Y, Du H, Qin Y, Activation of the signal transducers and activators of the transcription 3 pathway in alveolar epithelial cells induces inflammation and adenocarcinomas in mouse lung. Cancer Res, 2007, 67: 8494–8503 17875688 10.1158/0008-5472.CAN-07-0647

[19] Wu L, Du H, Li Y, Signal transducer and activator of transcription 3 (Stat3C) promotes myeloid-derived suppressor cell expansion and immune suppression during lung tumorigenesis. Am J Pathol, 2011, 179: 2131–2141 21864492 10.1016/j.ajpath.2011.06.028 PMC3181363

[20] Bromberg JF, Wrzeszczynska MH, Devgan G, Stat3 as an oncogene. Cell, 1999, 98: 295–303 10458605 10.1016/s0092-8674(00)81959-5

[21] Hevehan DL, Miller WM, Papoutsakis ET. Differential expression and phosphorylation of distinct STAT3 proteins during granulocytic differentiation. Blood, 2002, 99: 1627–1637 11861277 10.1182/blood.v99.5.1627

[22] Sasmono RT, Oceandy D, Pollard JW, A macrophage colony-stimulating factor receptor-green fluorescent protein transgene is expressed throughout the mononuclear phagocyte system of the mouse. Blood, 2003, 101: 1155–1163 12393599 10.1182/blood-2002-02-0569

[23] Lian X, Qin Y, Hossain SA, Overexpression of Stat3C in pulmonary epithelium protects against hyperoxic lung injury. J Immunol, 2005, 174: 7250–7256 15905571 10.4049/jimmunol.174.11.7250

[24] Wu L, Wang G, Qu P, Overexpression of dominant negative peroxisome proliferator-activated receptor-gamma (PPARgamma) in alveolar type II epithelial cells causes inflammation and T-cell suppression in the lung. Am J Pathol, 2011, 178: 2191–2204 21514433 10.1016/j.ajpath.2011.01.046 PMC3081168

[25] Qu P, Shelley WC, Yoder MC, Critical roles of lysosomal acid lipase in myelopoiesis. Am J Pathol, 2010, 176: 2394–2404 20348241 10.2353/ajpath.2010.091063 PMC2861104

[26] Qu P, Du H, Wilkes DS, Critical roles of lysosomal acid lipase in T cell development and function. Am J Pathol, 2009, 174: 944–956 19179613 10.2353/ajpath.2009.080562 PMC2665754

[27] Du H, Duanmu M, Witte D, Targeted disruption of the mouse lysosomal acid lipase gene: long-term survival with massive cholesteryl ester and triglyceride storage. Hum Mol Genet, 1998, 7: 1347–1354 9700186 10.1093/hmg/7.9.1347

[28] Lian X, Yan C, Yang L, Lysosomal acid lipase deficiency causes respiratory inflammation and destruction in the lung. Am J Physiol Lung Cell Mol Physiol, 2004, 286: L801–807 14644759 10.1152/ajplung.00335.2003

[29] Lian X, Yan C, Qin Y, Neutral lipids and peroxisome proliferator-activated receptor-{gamma} control pulmonary gene expression and inflammation-triggered pathogenesis in lysosomal acid lipase knockout mice. Am J Pathol, 2005, 167: 813–821 16127159 10.1016/s0002-9440(10)62053-6 PMC1698726

[30] Baddoo M, Hill K, Wilkinson R, Characterization of mesenchymal stem cells isolated from murine bone marrow by negative selection. J Cell Biochem, 2003, 89: 1235–1249 12898521 10.1002/jcb.10594

[31] Sackstein R, Merzaban JS, Cain DW, *Ex vivo* glycan engineering of CD44 programs human multipotent mesenchymal stromal cell trafficking to bone. Nat Med, 2008, 14: 181–187 18193058 10.1038/nm1703

[32] Anjos-Afonso F, Bonnet D. Nonhematopoietic/endothelial SSEA-1+ cells define the most primitive progenitors in the adult murine bone marrow mesenchymal compartment. Blood, 2007, 109: 1298–1306 17003364 10.1182/blood-2006-06-030551

[33] Qu P, Du H, Li Y, Myeloid-specific expression of Api6/AIM/Sp{alpha} induces systemic inflammation and adenocarcinoma in the lung. J Immunol, 2009, 182: 1648–1659 19155514 10.4049/jimmunol.182.3.1648 PMC2630116

[34] Sica A, Bronte V. Altered macrophage differentiation and immune dysfunction in tumor development. J Clin Invest, 2007, 117: 1155–1166 17476345 10.1172/JCI31422 PMC1857267

[35] Gabrilovich D. Mechanisms and functional significance of tumour-induced dendritic-cell defects. Nat Rev Immunol, 2004, 4: 941–952 15573129 10.1038/nri1498

[36] Ostrand-Rosenberg S, Sinha P. Myeloid-derived suppressor cells: linking inflammation and cancer. J Immunol, 2009, 182: 4499–4506 19342621 10.4049/jimmunol.0802740 PMC2810498

[37] Satoh M, Mioh H, Shiotsu Y, Mouse bone marrow stromal cell line MC3T3-G2/PA6 with hematopoietic-supporting activity expresses high levels of stem cell antigen Sca-1. Exp Hematol, 1997, 25: 972–979 9257811

[38] Rodriguez Mdel C, Bernad A, Aracil M. Interleukin-6 deficiency affects bone marrow stromal precursors, resulting in defective hematopoietic support. Blood, 2004, 103: 3349–3354 14701687 10.1182/blood-2003-10-3438

[39] Tanaka-Douzono M, Suzu S, Yamada M, Detection of murine adult bone marrow stroma-initiating cells in Lin(−)c-fms(+)c-kit(low)VCAM-1(+) cells. J Cell Physiol, 2001, 189: 45–53 11573203 10.1002/jcp.1141

[40] Gebe JA, Llewellyn M, Hoggatt H, Molecular cloning, genomic organization and cell-binding characteristics of mouse Spalpha. Immunology, 2000, 99: 78–86 10651944 10.1046/j.1365-2567.2000.00903.x PMC2327131

[41] Miyazaki T, Hirokami Y, Matsuhashi N, Increased susceptibility of thymocytes to apoptosis in mice lacking AIM, a novel murine macrophage-derived soluble factor belonging to the scavenger receptor cysteine-rich domain superfamily. J Exp Med, 1999, 189: 413–422 9892623 10.1084/jem.189.2.413 PMC2192994

[42] Li Y, Qu P, Wu L, Api6/AIM/Spalpha/CD5L overexpression in alveolar type II epithelial cells induces spontaneous lung adenocarcinoma. Cancer Res, 2011, 71: 5488–5499 21697282 10.1158/0008-5472.CAN-10-4225 PMC3156382

[43] Taraseviciene-Stewart L, Voelkel NF. Molecular pathogenesis of emphysema. J Clin Invest, 2008, 118: 394–402 18246188 10.1172/JCI31811 PMC2214683

[44] Alison MR, Lim S, Houghton JM. Bone marrow-derived cells and epithelial tumours: more than just an inflammatory relationship. Curr Opin Oncol, 2009, 21: 77–82 19125022 10.1097/CCO.0b013e32831de4cf

[45] Djouad F, Plence P, Bony C, Immunosuppressive effect of mesenchymal stem cells favors tumor growth in allogeneic animals. Blood, 2003, 102: 3837–3844 12881305 10.1182/blood-2003-04-1193

