# Supplementary material for: Myeloid-specific expression of Stat3C results in conversion of bone marrow mesenchymal stem cells into alveolar type II epithelial cells in the lung
Source: Sci China Life Sci. Author manuscript; Available in PMC 2021 Oct 21. (PMC8530440; doi:10.1007/s11427-012-4339-2)
Supplement: Supplementary material — Figure S1 The control study duplicating Figure 4D, except that doxycycline-untreated c-fms-rtTA/(TetO)7-CMV-Stat3C bitransgenic mice were used. Cell division of CFSE-labeled hSP-B 1.5-kb lacZ adherent BMSCs (+CFSE) was not detected in the bone marrow and spleen of recipient mice. [file NIHMS1740844-supplement-Supplementary_material.doc]

**Supporting Information** July 2012 Vol.55 No.7: 1

doi: 10.1007/s11427-012-4339-2


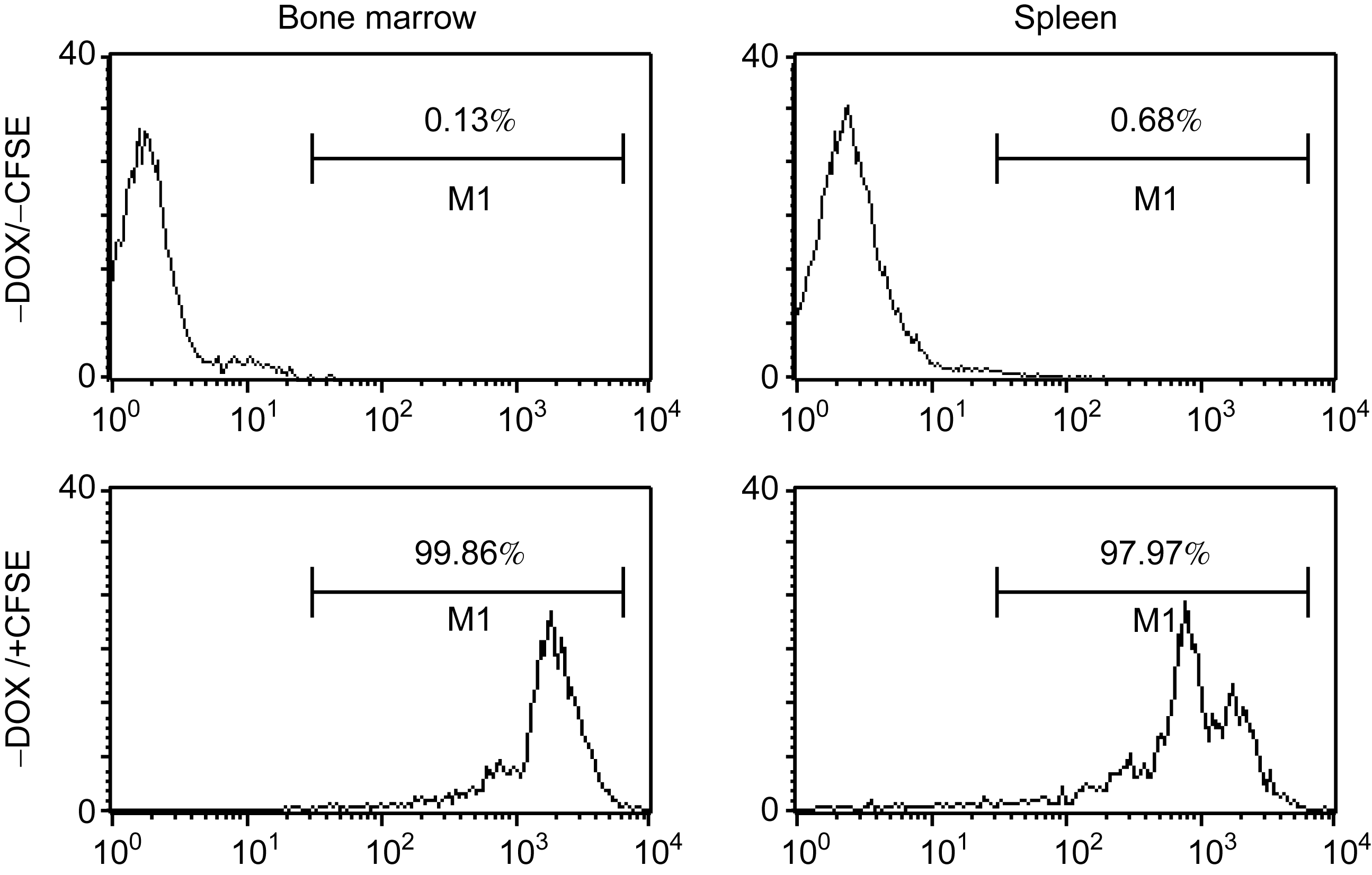


**Figure S1** The control study duplicating Figure 4D, except that doxycycline-untreated c-fms-rtTA/(TetO)_7_-CMV-Stat3C bitransgenic mice were used. Cell division of CFSE-labeled hSP-B 1.5-kb lacZ adherent BMSCs (+CFSE) was not detected in the bone marrow and spleen of recipient mice.
